# The Biological Activity of Propolis-Containing Toothpaste on Oral Health Environment in Patients Who Underwent Implant-Supported Prosthodontic Rehabilitation

**DOI:** 10.1155/2013/704947

**Published:** 2013-05-14

**Authors:** Tadeusz Morawiec, Arkadiusz Dziedzic, Iwona Niedzielska, Anna Mertas, Marta Tanasiewicz, Dariusz Skaba, Jacek Kasperski, Agnieszka Machorowska-Pieniążek, Marek Kucharzewski, Karolina Szaniawska, Włodzimierz Więckiewicz, Mieszko Więckiewicz

**Affiliations:** ^1^Department of Oral Surgery, Medical University of Silesia, Plac Akademicki 17, 41-902 Bytom, Poland; ^2^Department of Conservative Dentistry with Endodontics, Medical University of Silesia, Plac Akademicki 17, 41-902 Bytom, Poland; ^3^Department of Microbiology and Immunology, Medical University of Silesia, Jordana 19, 41-808 Zabrze, Poland; ^4^Department of Prosthetic Dentistry, Medical University of Silesia, Plac Akademicki 17, 41-902 Bytom, Poland; ^5^Department of Orthodontics, Medical University of Silesia, Plac Akademicki 17, 41-902 Bytom, Poland; ^6^Department of Descriptive and Topographic Anatomy, Medical University of Silesia, Jordana 19, 41-808 Zabrze, Poland; ^7^Division of Medicine and Dentistry, Department of Oral Surgery, Medical University of Warsaw, Ulica Nowogrodzka 59, 02-006 Warszawa, Poland; ^8^Department of Prosthetic Dentistry, Faculty of Dentistry, Wrocław Medical University, 50-425 Wrocław, Poland

## Abstract

The soft and periodontal tissues surrounding dental implants are particularly susceptible to bacteria invasion and inflammatory reactions due to complex histological structures. This study was carried out to investigate the influence of a propolis-containing hygienic agent on selected oral health parameters, oral microflora, and the condition of periodontal health. Sixteen subjects who underwent an oral rehabilitation with dental implants were selected and randomly assigned into two groups, which received a newly formulated propolis-containing toothpaste (3% (CA)) or a negative control without an active ingredient (CC). Approximal plaque index (API), oral hygiene index (OHI, debris component), and sulcus bleeding index (SBI) were assessed in three subsequent stages. During the first and last examinations, the swabs were employed for microbiological inoculation. Propolis-containing toothpaste was found to be distinctively effective in improving oral health and the occurrence of gingivitis triggered by dental plaque. The qualitative and quantitative changes in oral bacteria spectrum were observed. Antibacterial measures containing propolis might be used as a natural adjuvant to other active substances in individuals with a high risk of periodontal problems against pathogenic oral microflora.

## 1. Introduction

The bacterial plaque remaining over and under the gingiva contributes to the generation of inflammatory reaction in tissues surrounding teeth and dental implants which leads to the loss of collective tissue and alveolar bone attachment [1, 2]. Socransky et al. divided the microorganisms located on subgingival areas into five complexes. One of them, the so-called “red complex,” consisting of *Tannerella forsythensis*, *Porphyromonas gingivalis,* and *Treponema denticola,* is strongly associated with increased depth of periodontal and/or peri-implant pockets as well as bleeding when a probe is inserted [3]. Results of clinical and microbiological investigations also indicate that *Prevotella intermedia* or *Fusobacterium nucleatum, *among others, are potential etiological factors causing periodontitis and peri-implantitis problems [4]. Reduction of the number of those pathogenic micro-organisms could potentially influence the epidemiology of periodontium diseases, reducing their amount and intensification. The hygiene regime related to oral health maintenance is a critical area of implant-supported cases of oral rehabilitation. The periodontal tissues surrounding dental implants seem to be particularly susceptible to bacteria invasion and local inflammatory reactions [5–7]. 

Effective oral hygiene around dental implants can be challenging to achieve over the long term, and the patient, dentist, and dental hygienist must exercise considerable effort to achieve the desired results. In more recent years, implant maintenance and effective patient home care have been emphasized as critical factors needed for long-term success of dental implants [8]. As the number of patients treated with dental implants continues to grow, the patients must be fully aware of a proper maintenance with the use of preventive and therapeutic local measures [9, 10]. Among them, propolis—a resinous substance made by bees, possessing biological therapeutic activities—may play an important role in oral hygiene and in maintaining healthy soft tissues within the oral cavity. It is known for its ability to nourish periodontal tissues and prevent problems associated with gingiva and supported histological elements [8, 11]. 

Humans began using propolis more than 2000 years ago for many purposes, the foremost of which was applying it to wounds to fight infection. It promotes the healing process with its mild anti-infective properties [12]. Over time, it has been marketed in various forms, such as toothpastes, mouthrinses, and lozenges. Propolis extract is known to possess antimicrobial activity against *Streptococcus mutans,* a Gram-positive cocci, facultative anaerobic strains commonly found in the human oral cavity, and a significant contributor to plaque formation [13, 14]. Propolis has been also found beneficial in the treatment of gingivitis and oral mucosa lesions in case studies and pilot clinical studies [15, 16]. 

Over the last few decades, a worldwide trend has been observed in the use of natural products for pharmacological purposes due to their proven therapeutic effect. Propolis, which is a natural product widely consumed in the folk medicine since ancient times and a substance produced by the honeybees, seems to be a promising agent to be added to topical formulations due to its multidirectional properties [17]. Besides antioxidant and anti-inflammatory activity [18–21], epidemiological studies have detected that propolis has many beneficial properties, such as antibacterial, antifungal, antiviral, and antitumor [22–26]. Ethanolic extract of propolis (EEP) solutions has been used commercially on the market as toothpaste, mouth wash, lozenges, and so forth, as an effective antimicrobial and anti-inflammatory agent [27, 28]. However, it is still an underestimated ingredient in academic medicine and dentistry. In general, propolis is composed of 50% resin and vegetable balsam, 30% wax, 10% essential and aromatic oils, 5% pollen, and 5% various other substances, including organic debris depending on the place and time of collection [29]. The constituents of propolis vary widely due to climate, season, location, and year, and its chemical formula is not stable [30, 31]. Flavonoids—the main propolis ingredient—inhibit lipid peroxidation, platelet aggregation, capillary permeability and fragility, and the activity of enzyme systems including cyclo-oxygenase and lipoxygenase [28, 31]

The study aimed at investigating the local effect of the orally administered extract of propolis in the form of toothpaste on selected intraoral predictors representing oral health condition in the group of patients with dental implants-supported fixed and removable prosthodontic suprastructures.

## 2. Material and Methods

The clinical study was carried out to investigate the influence of a propolis-containing toothpaste on gingival health and the oral microbiota spectrum changes. This research was conducted in Oral Surgery Department, the Academic Centre of Dentistry and Specialist Medicine (Bytom, Poland) and Specialist Dental Clinic (Katowice, Poland) which were providing comprehensive dental care for the patients who underwent implant-prosthetic therapy. All implants were installed by the same operator, a specialist in oral surgery. The “oldest” installation was carried out 9 years ago, and the most recent was 2 years ago. The study was designed as a single blind two-group parallel study. The dental products and tests employed in the study were legal merchandises, commonly available and used. The study was not a clinical trial or a clinical experiment. 

### 2.1. Hygienic Compounds Preparation

Two samples of toothpastes, covered anonymously by a blank label and marked only with the letters (A) or (C), were compared: the tested toothpaste containing the active ingredient—3% ethanol extract of Brazilian propolis (CA, DentalPolisDX, “green” propolis), and a placebo as a control (CC), without active substance. Raw propolis was collected from the beekeeping section of the Seiri Alimentos Naturais, Brazil. Propolis samples were obtained from colonies of Africanized honeybees (*Apis mellifera*) in Minas Gerais State, Southeast Brazil and collected over a period of 2008 year from the plant *Baccharis dracunculifolia*, which is the main botanical source of the resin for the green propolis and determines the composition of Brazilian EEP rich in artepillin C [32]. The unprocessed propolis was sent to the Nihon Natural Therapy Co. Ltd. (Tokyo, Japan) for the preparation of the EEP ingredient. The toothpastes with 3% EEP and without of EEP (placebo) were prepared in Nippon Zettoc Co., Ltd., Tokyo, Japan.

### 2.2. Research Groups Inclusion Criteria

Subjects qualification for the study was based on medical and dental history, interview, and review of clinical records. The exclusion criteria from the investigation were as follows: lack of patient's valid consent, medically compromised patients, inability to comply with the follow-up visit requirements, patients receiving concurrent antibiotic treatment for any other purpose, individuals with confirmed adverse reactions to bee products, nursing or pregnant women, and recent postoperative oral surgery cases. Inclusion criteria comprised the age group of 22 to 65 years and patients free from systemic illness.

Sixteen subjects were randomly assigned into two groups of 8 subjects each, which received an unrecognized propolis-containing toothpaste (group A), or a negative control toothpaste (group C). Oral hygiene instructions were given in an attempt to improve their oral hygiene before entry into the study. The toothpastes were allocated according to groups to ensure balance. All subjects received professional advice regarding oral hygiene and were instructed to brush two times a day with the toothpaste recommended for at least two minutes and to refrain from all other oral hygiene measures until the next examination. 

Targeted groups of 16 patients who received implant-supported oral rehabilitation were examined in three subsequent stages: preliminary qualification at baseline (1st assessment), a follow-up after 7 days (2nd assessment), and after 8 weeks since the initial examination (3rd assessment). During the first and last examination, the swabs were used for microbiological examination of oral cavity microflora. The microbial material for smear tests was collected from the oral mucosa around fixed prosthetic suprastructures and peri-implant areas. Preliminary qualification of the subjects included past and current health problems, date of the last dental appointment, dental check-up intervals, dietary habits, carbonate-rich products intake, pattern and frequency of teeth brushing, and the use of additional domestic preventive measures (dental floss, mouthrinses, etc.). 

### 2.3. Clinical Examination Protocol

The selected, commonly used indices of oral health condition, API, OHI, and SBI, were assessed and scored at baseline, during follow-up visit and after eight weeks since the initial examination. Approximal plaque index (API, Lange & Ainamo, 1988) scored the presence of dental plaque in the interdental spaces, and oral hygiene index-debris component (OHI-D, Greene & Vermillion, 1960) was used for the assessment of amount of debris on all teeth and dental implants. For the aproximal plaque index, only the plaque (bacteria) in the spaces between the teeth was evaluated, in order to draw conclusions about the level of oral hygiene and individual risk of caries occurrence. Oral hygiene index determines the amount of soft debris or calculus on the four buccal surfaces of the selected teeth (dental implants): upper right first molar, upper right central incisor, upper left first molar, and lower central incisor and on the lingual surfaces of the lower left first molar and lower right first molar. The periodontal status (gingival health) was evaluated with the use of the modified sulcus bleeding index (SBI, Muhlemann-Son, 1971) and the recording of only “bleeding presence” or “bleeding absence” for all existing teeth or dental implants.

In order to assess the oral hygiene related to the fixed prosthodontics, the modified plaque index (MPI) was applied [33, 34] including the protocol adequate to OHI scores. MPI is valid for studies of peri-implant health in patients carrying mandibular implant overdentures on bars as long as the hygiene in abutments as in bars is evaluated as much. The lack of the bar's hygiene assessment would suppose an allocation of plaque values lower to real ones, so a poor hygiene would be undervalued. These patients were included into an oral hygiene protocol to avoid subsequent peri-implant complications as mucositis or peri-implantitis. MPI scores are as follows: score 0—no plaque, score 1—no plaque at first sight, presence when slipping probe on abutment and/or on bar, score 2—moderate plaque at first sight on abutment and/or on bar, and score 3—plaque at first sight, abundant, that occupies more than 1/3 of abutment and/or bar.

Certain mild degree of gingival recessions around dental implants was noted during examinations; however, these observations did not meet the criteria of periimplantitis or other substantial peri-implant pathology. The mid-term and long-term success rates of dental implants were fully predictable, including good clinical prognosis. None of the signs indicating the possibilities of future implants failure was observed. The initial clinical examination of stabilization of dental implants and prosthodontic restorations was carried out and was graded as “within acceptable limits.”

Additionally, the questionnaire containing the questions about organoleptic and rheological properties of toothpastes was filled in by all patients in order to gather additional information. The questions taken into individual evaluation included color, taste, smell, foaming ability, and general opinion about tooth brushing effectiveness (tooth smoothness, freshness feeling), and they were assessed in a 3-grade scale. The data was then subjected to statistical analysis. Patients were assessed by two examiners using the same technique and procedure in order to validate the objective clinical scores. All bacterial samples were taken in the same manner, applying the standard protocol for oral microflora inoculums.

### 2.4. Bacterial Strains Isolation and Microbiological Investigation

The estimation of bacterial spectrum was performed in quantitative and qualitative manner using standard methods for microbiological inoculation. A number of 16 subjects who participated in the study were scraped the gingiva margins surrounding dental implants with a sterile swab by the clinician. Samples were collected by two examiners using the same procedure. The vials were delivered within a single working day and then immediately seeded in the laboratory within 24 hours, allowing the bacteria strains isolation from clinical specimens, which were subject for further inoculation. 

The biological material collected for microbiological investigation was cultured on a suitable medium (Columbia agar, Schaedler K3 agar, Sabouraud agar) by bioMerieux (Marcy l'Etoile, France). Aerobic bacteria were multiplied on solid medium Columbia agar, with 5% addition of ram blood, in the temperature of 37°C. Anaerobic bacteria were multiplied on solid medium Schaedler K3, with 5% addition of ram blood, in the temperature of 37°C in anaerobic conditions, with the use of GENbag anaer by bioMérieux (Marcy l'Etoile, France). Fungi of the *Candida *family, on the other hand, were multiplied on selective solid medium Sabouraud agar, in the temperature of 35°C in aerobic conditions. 

After they had been isolated and cultured further, each of the microorganisms was identified as regards its species, using the following set of reagents: Api 20 E, Api 20 NE, and Api Candida by BioMérieux (Marcy l'Etoile, France); ENTEROtest 24 N, and NEFERMtest 24 N, STREPTOtest 24, STAPHYtest 24, ANAEROtest 23 by Erba-Lachema (Brno, Czech Republic). 

The data of individual patients were treated as confidential and were not identifiable in any publication that emerged in relation to the examination. The study represented a separated part of the main research project of Medical University of Silesia, supported by KNW-2-102/10 SUM grant. The research project was granted by the decision of Bioethics Committee of the Medical University of Silesia (decision no. 6/2010)

### 2.5. Statistical Analysis

Nonparametric tests were applied for statistical verification of assumed research hypotheses and analysis of quantitative and qualitative data (OHI, API, SBI, toothpaste features). The comparison of the two dependent groups was carried out using nonparametric Wilcoxon signed-rank test (OHI, SBI, API). For the two independent samples the nonparametric Mann-Whitney *U*-test (OHI, SBI, API) was applied for comparing pairs of groups. Three dependent groups were compared with the use of Friedman ANOVA test. All tests applied were two tailed, and a *P*-value of ≤0.05 was considered statistically significant. Statistica 9.0 software (StatSoft, USA) was used for statistical analysis (Medical University of Silesia licence, Katowice, Poland). 

## 3. Results

From the total group of 21 patients, 16 successfully completed the study according to the research protocol (10 women and 6 men). The restricted number of subjects with fitted dental implants supporting prosthetic restorations reflected the strict inclusion criteria for the study.  Table 1 presents the implant-prosthetic profile of the examined groups in terms of the number of dental implants installed and the type of prosthetic suprastructure (crown, bridge, overdenture) supported by endosseous implants. The total number of single endosseous, titanium-made implants installed for the group (A) and (C) was 29 and 24, respectively. One patient from the group (A) was using the bar-retained removable overdenture, and one subject from the group (C) had an overdenture supported by two-single implants and locators. Vast majority of examined patients were the users of single porcelain-to-metal fused crowns retained by to the single endosseous implants with conical metal abutments and active threads.

### 3.1. Oral Health Conditions

**Figure 1:**
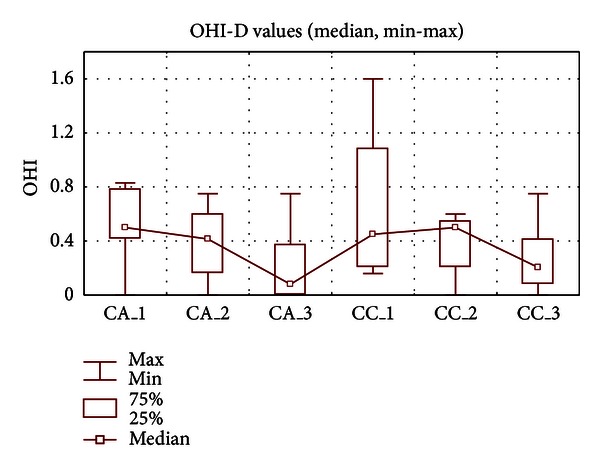
Oral hygiene index values (median, min-max) for examined groups A and C.

The overall distribution of the range of Greene and Vermilion's oral hygiene index (OHI-D, debris component) scores in the evaluated groups is shown in Table 2. For one subject from groups (A) and (C) carrying mandibular implant-retained overdenture on bar, the modified index of oral hygiene was applied [34]. The OHI-D index (median) for (A) and (C) groups was established as 0.2 and 0.08, respectively, and the differences were not statistically significant comparing both mentioned groups after 1st, 2nd, and 3rd examination (Figure 1). OHI-D value (median) of the group (A), which consisted of the patients using propolis-containing toothpaste, decreased significantly after 8 weeks of the study (*P* < 0.05). On the other hand, the influence of toothpaste CC was not statistically significant, despite of the OHI-D scores decreasing tendency at the end of the study and the “*P*” value close to 0.05 (*P* = 0.061). It may be explained by the effect of patients participation in the study, the use of specific hygienic agents alone, and the improved toothbrushing following oral hygiene instructions, which could influenced the obtained results, irrespective of the presence or absence of a propolis additive.

**Figure 2:**
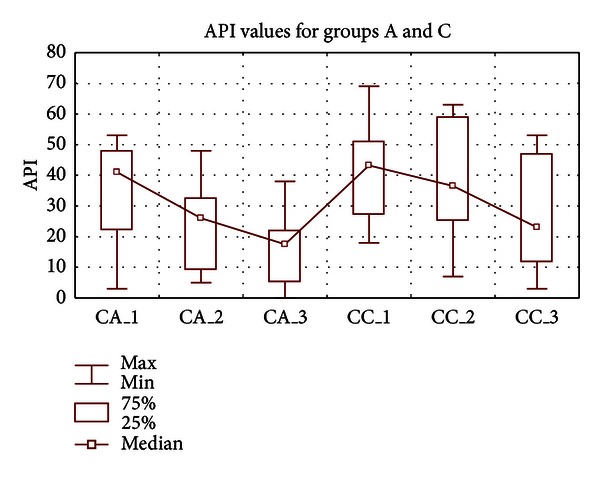
Approximal plaque index values (median, min-max) for examined groups (A) and (C).

The decreasing tendency of the Lange API scores was also observed within the assessed groups (A) and (C), and the significant differences were noted for group (A) only, essentially between the first and the last clinical assessment (*P* < 0.05). During the first visit, the average oral hygiene profile of patients from group (A) was classified as an “average oral hygiene” (62.5%) while at the end of the study the mean API score was determined as “optimal hygiene” (87.5%). Vast majority of the patients from group (C) represented an "average oral hygiene" and “quite good oral hygiene” profile during the first and second follow-up assessments, respectively (Table 3, Figure 2).

The third assessment of the periodontal tissues carried out using Muhlemann-son's sulcus Bleeding index allowed to classify all the patients from group (A) as the subjects described as “normal, healthy gingiva, no bleeding” with SBI value <10% (Table 4). However, the differences between the groups (A) and (C) were not statistically significant after 1st, 2nd and 3rd assessment (*P* > 0.05, Mann-Whitney *U*-test). A significant decrease of SBI value during the last follow-up assessment was observed for the group (A) patients (*P* < 0.05). Patients belonging to the group (C) (50%, *n* = 4) who used the CC toothpaste were initially qualified into the subjects with slight, initial gingivitis (bleeding on probing, without shape and color changes of gingiva) which required oral hygiene instructions and hygienic regime. 

### 3.2. Patients Opinions Analysis Based on Questionnaire

The questionnaire-based assessment of the rheological and organoleptic properties of the investigated toothpastes as well as the patients individual opinions regarding a comfort when using these preparations revealed differences between the groups A and C. The taste and smell were assessed with higher scores, while the main complaint was pointed out towards the foaming ability, which was scored as “unsatisfactory” by 62.5% of patients (*n* = 5) from group A and as only “satisfactory” by 62.5% of patients from group C (*n* = 5). The colour of the toothpaste CA was also not graded well, and only one patient from group A rated it as “good.” Generally, the CC toothpaste did not evoke such subjective opinions and was better accepted (Table 5).

### 3.3. Microbiological Identification of Peri-Implant Oral Microbiota

Total amount of isolated microorganisms present from peri-implant areas of the patients using toothpaste CA and CC is stated according to Table 6. The results of microbiological examinations of the clinical specimens demonstrated a substantial quantitative as well as qualitative differentiation of the oral cavity microflora composition in patients with dental implants (group A) who were using implant-supported prosthetic appliances, applying toothpaste with EEP (CA preparation) for oral hygiene maintenance a few times per day. The microbiological material collected before the use of propolis-containing toothpaste (first assessment) allowed to identify a total of 16 isolates of microorganisms' representing 10 species/strains in the group of patients applying CA toothpaste with active organic ingredient.

Second microbiological assessment, performed after eight weeks since the study commencement, revealed a total of 32 isolates of microorganisms, representing 15 species (Table 7). In the patients of group (A), four species of microorganisms were eliminated (*Klebsiella oxytoca, Serratia liquefaciens, Staphylococcus epidermidis *MSCNS, *Sarcina *sp.); at the same time, the physiological and transient micro-flora of oral cavity has been enriched by the addition of 9 new species of microorganisms: *Streptococcus sanguinis, Streptococcus vestibularis, Streptococcus acidominimus, Ruminococcus productus, Veillonella parvula, Bifidobacterium adolescentis, Bifidobacterium dentium, Actinomyces naeslundii,* and *Citrobacter freundii. *The second assessment of the (A) group of patients also revealed a less numerous presence of *Escherichia coli* isolates than the first examination did. At the same time, the second examination revealed the presence of more isolates of the bacteria *Streptococcus mitis*, *Streptococcus salivarius, Neisseria *species, and *Candida* fungi. During the first examination, *Candida albicans* has been isolated in one person; while *Candida famata *in another person, whereas in the case of the second examination, three isolates of *Candida albicans* have been found (subjects different than the case of 1st examination) as well as one isolate of *Candida famata* (the same subject as in 1st examination). These findings can be related to the improved and excessive daily oral hygiene, following the thorough instruction given during first examination. 

Considering the microbiological profile in the group of eight patients who were using toothpaste without EEP (group C) for oral cavity hygiene, after eight weeks of application of CC preparation a similar count of microorganisms isolates was observed (Table 8). The material collected before the toothpaste CC application commencement (1st examination) was represented by a total of 23 isolates of microorganisms belonging to 13 species. As a result of the second examination, carried out after eight weeks since CC application commenced, a total of 28 isolates of microorganisms allocated to 16 species have been obtained. The second microbiological examination of group (C) patients revealed the elimination of 7 species of microorganisms: *Streptococcus sanguinis, Escherichia coli, Enterobacter aerogenes, Enterobacter amnigenus, Mitsuokella multiacidus, Serratia liquefaciens,* and *Serratia grimes. *After the period of 8 weeks the oral cavity micro-flora got enriched with 10 new species of physiological flora microorganism and potential pathogens: *Streptococcus vestibularis, Streptococcus *β* haemolyticus F group, Staphylococcus epidermidis *MSCNS*, Sarcina *sp.*, Veillonella parvula, Bifidobacterium adolescentis, Klebsiella oxytoca, Klebsiella pneumoniae, Pseudomonas aeruginosa,* and *Serratia marcescens.* The findings of the second examination, concerning the patients marked as a group (C), also showed a higher count of isolated *Streptococcus mitis *and isolates of the *Neisseria* species as well as fungi of the *Candida* family (*Candida albicans, Candida glabrata*).

## 4. Discussion

Individual home care and consistent professional maintenance have proven to be critical to the success and longevity of endosseous dental implants. This is especially true in an environment with adjacent natural teeth, which if affected by periodontal disease could act as a reservoir for pathogenic bacteria and seed the peri-implant sulcus [35, 36]. An implant patient's home care regimen should be individually tailored according to each patient's needs. These needs are based on the location and angulation of the dental implants, the position and length of transmucosal abutments, the type of prosthesis, and the rate of plaque and calculus accumulation [37]. Proper monitoring and maintenance are essential to ensure the longevity of the dental implant through a combination of appropriate professional care, evaluation, and effective patient oral hygiene. Oral rinses with antimicrobial properties, containing essential oils or chlorhexidine, have been advocated for use in patients with implants [38]. In this area, the routine use of propolis-containing toothpaste seems to have a beneficial effect on peri-implant tissues and plaque accumulation. In a recent study, authors Tanasiewicz et al. [16] demonstrated that hygienic experimental preparations (toothpaste and gel) containing 3% ethanol propolis extract efficiently support reduction of dental plaque and have the therapeutic local effect on marginal periodontium. These results are coherent to our findings and observations, based on the SBI values, constituting the conclusion that domestic products for oral hygiene support the antiplaque action and have the anti-inflammatory effect on marginal periodontium. The proven anti-inflammatory action of propolis seems to be particularly advantageous for prophylactic procedures of the patients with dental implants and increased risk of periodontal inflammatory problems, that is, gingivitis and chronic periodontitis [39–41].

Current trend reveals a return to complementary medicine and alternative treatment methods, due to the developing resistance to modern medications and antibiotics. Only few studies have investigated the activity of propolis ethanolic extract towards oral pathogens, particularly periodontopathic bacteria [36, 38, 39, 42]. Propolis samples were found to be active mainly against Gram-positive bacteria and some fungi. They presented also a weak activity against Gram-negative bacteria [14, 27, 41, 42]. The pharmacologically active constituents against oral bacteria in Brazilian propolis are flavonoids (flavones, flavonols, flavanones), phenolics, and aromatics, including *p-*coumaric acid, ferulic acid, cinnamic acid, and its derivative—drupanin, baccharin, and artepillin C, chrysin—tectochrysin, pinocembrin, pinobanksin, isosakuranetin, kaempferol, kaempferide, and quercetin [43–45]. 

In our study, the analysis of the influence of toothpaste containing 3% ethanol extract of propolis upon the mouth cavity micro-flora revealed beneficial quality changes in its species composition, consisting mainly of elimination of potential bacteria pathogens, particularly *Enterobacteriaceae *family rods. To summarize the results of microbiological examinations, it can be stated that the propolis included in toothpaste, thanks to its antimicrobial properties, has beneficial influence upon the modification of oral cavity bacterial micro-flora, whereas it hardly influences fungi of the *Candida *family. The increase of the *Candida* strains during the second assessment may be caused by intensive oral hygiene regime and hygienic habits improvement. The application of propolis in preparations used for routine oral cavity hygiene allows to eliminate microorganisms that are pathogens, as well as microorganisms of physiological flora, listed among opportunistic pathogens. The European Patent EP 1738781 A2 describes the method which involves using propolis as coating material for medical implants [46].

Clinical researches have examined the association between oral microorganisms which are found in the saliva as non-adhering populations and as plaque, a microbial biofilm, and specific oral conditions such as dental caries, periodontal disease, and oral mucosa diseases [5, 6, 36, 39, 41, 47]. Koo et al. stated that mouthrinses containing propolis showed significant reduction of dental plaque and also inhibition of bacterial polysaccharides formation [47]. Similar to these findings, we assumed that the choice of specific therapeutic product including propolis-containing toothpaste may have a direct influence on oral hygiene regime improvement and elimination of hygiene negligence in case of healthy patients or with minor periodontal problems. Propolis constitutes a natural alternative which helps to maintain oral health and healthy periodontium.

Study accomplished by Dodwad and Jha Kukreja [48] evaluated the effect of propolis mouthwash on plaque accumulation and gingivitis of a group of subjects who completely stopped the oral hygiene regime, that is, toothbrushing. Plaque index increased on the 5th day, with the estimated value for propolis 68% and 16% for chlorhexidine. Moreover, propolis revealed 7% and chlorhexidine 9% increase in gingival index. These results suggested that propolis is not better than chlorhexidine in reducing plaque formation but may be more efficient in reduction of gingival inflammation. This is in accordance with studies by Murray et al., 1997 [49]. The research reported by Koo et al. indicated substantial effectiveness of propolis extract in inhibiting the growth of bacteria that belong to the “red complex" [50]. Santos et al., Feres et al., and Koru et al. also confirmed the antibacterial effectiveness of propolis extract towards the pathogens causing periodontitis [5, 6, 26, 51, 52]. Different groups of organic compounds have been identified from propolis, mainly flavonoids and phenolic acids (esters), which are responsible for many of the biological activities attributed to European, Brazilian, and Asian propolis [53–55]. 

Due to the inhibition of the development of pathogens causing parodontitis/peri-implantitis, extracts of propolis for mouth rinsing, or toothpastes based on propolis extract seems to be a promising agent, not only for prophylaxis but also for the treatment [5, 6]. Bruschi et al. demonstrated that the therapeutic propolis-containing gel, applied locally to gingival pockets may be effective for the treatment of periodontal diseases [5, 6, 56]. The findings of Coutinho's microbiological study revealed that subgingival irrigations with the propolis extract, applied in periodontal course of treatment, improved the treatment outcomes more significantly than scaling and root planning alone [57]. The prophylactic action of propolis towards periodontal tissues, as an additive to mouths rinses or toothpastes, allows to reduce the dental plaque formation and the initial signs of gingivitis [58]. Numerous studies have also proven immunomodulatory action of propolis extract [59], among the other pharmacological activities, such as inhibitory action towards bacterial biofilm formation [60].

The general improvement of oral hygiene which was observed during the study can be partially related to “subject of investigation effect.” Patients taking part in any clinical research focusing on oral hygiene adjust and improve their brushing habits and carry them out more efficiently than routinely. Our findings indicate that propolis and/or its compounds are promising antibacterial agents for prevention of oral diseases. The effective biological action observed for the propolis extract suggests its usage as an adjuvant to, for example, therapy of periodontal problems. The results of the presented clinical study may suggest a positive influence of propolis-containing toothpaste (3% EEP) in patients with the occurrence of gingivitis caused by dental plaque. The routine daily use of propolis-containing hygienic measures seems to have a beneficial effect on peri-implant tissues by reducing a plaque accumulation and preventing development of chronic periodontal disease.

A further step should be given to verify if a dose sufficient to eradicate the target microorganisms can be reached within the subgingival environment without causing adverse effects, over a long-term period of use. However, the representative group containing more individuals with dental implants is needed for relevant evaluation. The local hygienic and domestic agents (e.g., toothpastes) containing natural products need to have the appropriate and improved organoleptic features, including more accepted colour and foaming action. It needs to be emphasised that when using propolis-based hygienic preparations, the patient needs to be aware and needs to be informed about potential occurrence of side effects, particularly possible allergic reactions [16].

## 5. Conclusion

This study indicates a positive biological activity of propolis-containing toothpaste with respect to the oral microbiota spectrum. The results obtained suggest that propolis might be used as a natural alternative or additive to chemical mouthwashes, for example, chlorhexidine in individuals suffering from periodontal problems associated with implants usage. Although further and long-term trials are required for more conclusive evidence, antibacterial measures containing propolis would be promising local agents acting against pathogenic oral microflora. 

## Figures and Tables

**Table 1:** Total number of dental implants installed and the type of prosthetic suprastructure (crown, bridge, overdenture) supported by endosseous implants in each group (A) and (C).

	Group (A)	Group (C)
Age (mean)	49.50	45.87
Gender		
Male	2	4
Female	6	4
Number of single dental implants	29	24
Mean number of dental implants per patient	3.62	3
Number of other abutments (bars)	1	0
Number of single crowns	16	13
Number of fixed suprastructures (at least 3 pointic)	4	3
Number of implant-supported overdentures	1	1

**Table 2:** OHI debris component—assessment for the group (A) and group (C) (% of cases within the range and min-max value).

	Oral hygiene assessment (OHI-debris)						Friedman ANOVA test (*P*)	Wilcoxon rank test (*P*)
	1st	
Group (C)	0.0–0.5	62.5%	0.0–0.5	75.0%	0.0–0.5	87.5%	0.061	(1) : (2) n/a (2) : (3) n/a (1) : (3) n/a
	0.6–1.0	12.5%	0.6–1.0	12.5%	0.6–1.0	12.5%		
	1.1–2.0	25.0%	1.1–2.0	12.5%	1.1–2.0	0.0%		
	(0.16–1.6)		(0–1.25)		(0–0.75)	

Group (A)	0.0–0.5	62.5%	0.0–0.5	75.0%	0.0–0.5	87.5%	0.003*	(1) : (2) = 0.027* (2) : (3) = 0.027* (1) : (3) = 0.043*
	0.6–1.0	37.5%	0.6–1.0	25.0%	0.6–1.0	12.5%		
	1.1–2.0	0.0%	1.1–2.0	0.0%	1.1–2.0	0.0%		
	(0–0.83)		(0–0.75)		(0–0.75)	

Mann-Whitney *U* test (*P*)	0.958		0.792		0.494		—	—

*Statistically significant *P* value < 0.05.

**Table 3:** API ranges—assessment for the group (A) and group (C) (% of cases).

	Oral hygiene assessment (interproximal spaces)						Friedman ANOVA test (*P*)	Wilcoxon rank test (*P*)
	1st		2nd		3rd	
Group (C)	Optimal	25.0%	Optimal	12.5%	Optimal	50.0%	0.149	(1) : (2) n/a (2) : (3) n/a (1) : (3) n/a
	Quite good	12.5%	Quite good	50%	Quite good	12.5%		
	Average	50%	Average	37.5%	Average	37.5%		
	Bad	12.5%	Bad	0.0%	Bad	0.0%		

Group (A)	Optimal	25.0%	Optimal	50.0%	Optimal	87.5%	0.005*	(1) : (2) = 0.201 (2) : (3) = 0.022* (1) : (3) = 0.456
	Quite good	12.5%	Quite good	37.5%	Quite good	12.5%		
	Average	62.5%%	Average	12.5%	Average	0.00%		
	Bad	0.0%	Bad	0.00%	Bad	0.00%		

Mann-Whitney *U* test (*P*)	0.472		0.109		0.183		—	—

*Statistically significant *P* value < 0.05.

**Table 4:** SBI—assessment for the group (A) and group (C) (% of cases).

	Periodontal assessment						Friedman ANOVA test (*P*)	Wilcoxon rank test (*P*)
	1st		2nd		3rd	
Group (C)	Normal gingiva SBI < 10%	50%	Normal gingiva SBI < 10%	62.5%	Normal gingiva SBI < 10%	62.5%	0.483	(1) : (2) n/a (2) : (3) n/a (1) : (3) n/a
	Bleeding on probing	50%	Bleeding on probing	37.5%	Bleeding on probing	37.5%		

Group (A)	Normal gingiva SBI < 10%	50%	Normal gingiva SBI < 10%	75%	Normal gingiva SBI < 10%	100%	0.015*	(1) : (2) = 0.067 (2) : (3) = 0.043* (1) : (3) = 0.874
	Bleeding on probing	50%	Bleeding on probing	25%	Bleeding on probing	0.0%		

Mann-Whitney *U* test (*P*)	0.599		0.344		0.127		—	—

*Statistically significant *P* value < 0.05.

**Table 5:** Descriptive scale of tested toothpastes properties (CA and CC).

		Subjects number (%)	
Toothpaste features			
		Group A (CA)	Group C (CC)
Taste	Unsatisfactory	1 (12.5%)	0 (0.0%)
	Satisfactory	1 (12.5%)	0 (0.0%)
	Good	6 (75.0%)	8 (100%)

Smell	Unsatisfactory	2 (25.0%)	0 (0.0%)
	Satisfactory	1 (12.5%)	2 (25%)
	Good	5 (62.5%)	6 (75.0%)

Colour	Unsatisfactory	5 (62.5%)	0 (0.0%)
	Satisfactory	2 (25.0%)	2 (25.0%)
	Good	1 (12.5%)	6 (75%)

Foaming	Unsatisfactory	5 (62.5%)	1 (12.5%)
	Satisfactory	0 (0.00%)	5 (62.5%)
	Good	3 (37.5%)	2 (25.0%)

Cleaning ability	Unsatisfactory	2 (25.0%)	0 (0.0%)
	Satisfactory	6 (75.0%)	0 (0.0%)
	Good	0 (0.0%)	8 (100%)

**Table 6:** Total amount of isolated microorganisms isolated from periimplant areas in the patients using toothpastes CA and CC.

		Micrococci Gram (+)	Micrococci Gram (−)	Rods Gram (+)	Rods and bacilli Gram (−)	Fungi	Total
Group (A)	Test 1	6	2	0	6	2	16
	Test 2	14	9	3	2	4	32

Group (C)	Test 1	9	5	0	7	2	23
	Test 2	12	7	1	4	4	28

**Table 7:** Microorganisms isolated from oral cavity samples; the investigated group (A) of patients with dental implants (*n* = 8) and implant-retained prosthetic suprastructures who were using propolis-containing toothpaste (CA).

Isolated microorganisms (strains)	Baseline examination									Final examination								
	1	2	3	4	5	6	7	8	all	1	2	3	4	5	6	7	8	all
Gram-positive cocci																		
* Streptococcus mitis *			x		x		x		**3**	x	x		x		x		x	**5**
* Streptococcus sanguinis *									—				x					**1**
* Streptococcus salivarius *						x			**1**			x		x		x	x	**4**
* Streptococcus vestibularis *									—						x			**1**
* Streptococcus acidominimus *									—	x					x			**2**
* Staphylococcus epidermidis *MSCNS			x						**1**									—
* Ruminococcus productus *									—				x					**1**
* Sarcina *sp.						x			**1**									—
Gram-negative cocci																		
* Neisseria *spp.					x	x			**2**	x		x	x	x	x	x	x	**7**
* Veillonella parvula *									—			x		x				**2**
Gram-positive Actinobacteria																		
*Bifidobacterium adolescentis *									—								x	**1**
* Bifidobacterium dentium *									—			x						**1**
* Actinomyces naeslundii *									—			x						**1**
Gram-negative bacilli (rod-shaped)																		
*Citrobacter freundii *									—		x							**1**
* Escherichia coli *		x					x	x	**3**					x				**1**
* Klebsiella oxytoca *					x				**1**									—
* Serratia liquefaciens *	x			x					**2**									—
Fungi																		
* Candida albicans *						x			**1**		x			x		x		**3**
* Candida famata *			x						**1**			x						**1**

Total isolated strains	**1**	**1**	**3**	**1**	**3**	**4**	**2**	**1**	**16**	**3**	**3**	**6**	**4**	**5**	**4**	**3**	**4**	**32**

x: the presence of bacterial strain in investigated material.

**Table 8:** Microorganisms isolated from oral cavity samples; the investigated group (C) of patients with dental implants (*n* = 8) who were using toothpaste (CC) without EEP.

Isolated microorganisms (strains)	Baseline examination									Final examination								
	1	2	3	4	5	6	7	8	all	1	2	3	4	5	6	7	8	all
Gram-positive cocci																		
* Streptococcus mitis *						x	x		**2**		x	x		x			x	**4**
* Streptococcus sanguinis *	x		x				x		**3**									—
* Streptococcus salivarius *		x			x				**2**						x	x		**2**
* Streptococcus vestibularis *									—	x								**1**
* Streptococcus haemolyticus F group *									—					x				**1**
* Staphylococcus aureus* MSSA		x				x			**2**		x						x	**2**
* Staphylococcus epidermidis* MSCNS									—					x				**1**
* Sarcina *sp.									—	x								**1**
Gram-negative cocci																		
* Neisseria *spp.	x		x		x	x	x		**5**	x	x	x		x	x	x		**6**
* Veillonella parvula *									—								x	**1**
Gram-positive Actinobacteria																		
* Bifidobacterium adolescentis *									—					x				**1**
Gram-negative bacilli (rod-shaped)																		
* Escherichia coli *				x					**1**									—
* Enterobacter aerogenes *								x	**1**									—
* Enterobacter amnigenus *			x						**1**									—
* Klebsiella oxytoca *									—				x					**1**
* Klebsiella pneumoniae *									—						x			**1**
* Mitsuokella multiacidus *						x	x		**2**									—
* Pseudomonas aeruginosa *									—				x					**1**
* Serratia liquefaciens *	x								**1**									—
* Serratia grimesii *				x					**1**									—
*Serratia marcescens *									—		x							**1**
Fungi																		
*** *** *Candida albicans *	x								**1**	x	x							**2**
* Candida glabrata *					x				**1**					x			x	**2**

Total isolated strains	**4**	**2**	**3**	**2**	**3**	**4**	**4**	**1**	**23**	**4**	**5**	**2**	**2**	**6**	**3**	**2**	**4**	**28**
